# Effects of Dietary Vitamin E Supplementation on Reproductive Performance, Egg Characteristics, Antioxidant Capacity, and Immune Status in Breeding Geese during the Late Laying Period

**DOI:** 10.3390/antiox11102070

**Published:** 2022-10-20

**Authors:** Zhenming Fu, Tao Zhong, Xiaoli Wan, Lei Xu, Haiming Yang, Houming Han, Zhiyue Wang

**Affiliations:** 1 College of Animal Science and Technology, Yangzhou University, Wenhui East Road 48#, Yangzhou 225009, China; 2 Jiangsu Lihua Animal Husbandry Co., Ltd., Changzhou 213000, China; 3 Joint International Research Laboratory of Agriculture and Agri-Product Safety of Ministry of Education of China, Yangzhou University, Yangzhou 225009, China

**Keywords:** vitamin E, goose, reproductive performance, egg characteristics, antioxidant capacity, immune status

## Abstract

This study aimed to tentatively evaluate the effects of dietary vitamin E (VE) on goose reproductive physiology through the investigation of reproductive performance, egg characteristics, antioxidant capacity, and immune status in breeding geese. A total of 480 female and 96 male Jiangnan White breeding geese were randomly assigned to four treatments with four replicates, and each replicate had 30 females and six males. Four levels of VE were successively added to four treatment diets from 48 to 54 weeks of age, representing the effects of VE deficiency (0 IU/kg), basic-dose VE (40 IU/kg), middle-dose VE (200 IU/kg), and high-dose VE (2000 IU/kg). Neither the egg-laying rate nor the healthy-gosling rate were affected by any of the VE supplementations (*p* > 0.05). The qualified egg rate, hatchability of fertilized eggs, and spleen index were increased by each VE supplementation (*p* < 0.05). Egg fertility, the concentration of plasma reproductive hormones (i.e., the follicle-stimulating hormone, estradiol, and progesterone), follicular development, and antioxidant enzyme activities—i.e., the concentration of malondialdehyde (MDA), total antioxidant capacity (T-AOC), superoxide dismutase (SOD), and glutathione peroxidase (GSH-Px)—in the liver and ovary were improved by 200 IU/kg of dietary VE (*p* < 0.05). Plasma VE concentration, immunoglobulin A, and immunoglobulin G content were increased, whereas plasma vitamin D_3_ concentration was reduced by increasing dietary VE levels to 2000 IU/kg (*p* < 0.05). The VE deposition of yolk, the yolk color depth, and the albumen rate were increased by each VE supplementation (*p* < 0.05). Antioxidant enzyme activities (i.e., MDA concentration, T-AOC, SOD, and GSH) in yolk were improved by 200 IU/kg and 2000 IU/kg of dietary VE (*p* < 0.05), compared with 0 IU/kg. The VE deposition was significantly correlated with GSH activity and the MDA concentration in egg yolk (*p* < 0.05). However, the high intake of dietary VE (2000 IU/kg vs. 200 IU/kg) decreased egg fertility (*p* < 0.05) and reduced the antioxidant capacity in the liver and ovary (*p* < 0.05). The qualified egg rate was positively correlated to immunoglobulin production (*p* < 0.05). Egg fertility and hatchability were correlatively improved by increased antioxidant enzyme activity; decreased MDA in the liver and ovary; hatchability; and enhanced immune status (*p* < 0.05). To sum up, both VE deficiency and high-dose VE (2000 IU/kg) reduced reproductive performance, whereas a dose of 200 IU/kg VE achieved optimal fertility, possibly through enhancing antioxidant capacity and immune status.

## 1. Introduction

Fertility function is critical for the successful production of healthy offspring in poultry. However, oxidant stress and immunological imbalance may hasten the decline of reproductive performance in breeding poultry of advancing age [1,2]. Supplementation of antioxidants in diets has become a key strategy to solve this problem in poultry reproduction.

For one hundred years, vitamin E (VE) has been extensively studied as a substance that is necessary in reproduction [3]. Poultry does not synthesize enough VE and depends on dietary sources to meet daily requirements [4]. The influence of VE deficiency and the nutritional demand for VE has been comprehensively investigated in breeding poultry, including chickens, ducks, and quails [5,6,7]. Specifically, an appropriate VE supplementation can maintain reproductive performance and egg quality, which are the essential criteria for eventual profitability. In recent years, geese production has grown rapidly and created substantial economic benefits [8]. By summarizing the relevant data in China, Wang [9] concluded that 30 IU/kg to 50 IU/kg of VE could meet the basic VE requirement of breeding geese. In the latest studies, 40 IU/kg of VE was supplemented to formulate the diets of breeding geese [10,11]. 

However, to achieve higher performance, VE supplementation in poultry diets has been tentatively increased in intensive commercial production. Higher dietary VE additions, including 60, 100, 120, 160, and 200 IU/kg [5,12,13,14,15,16], were reported to obtain better reproductive performance and antioxidant capacity in breeding chickens, compared with basic additions. However, the effects of VE addition beyond the basic demand have not been thoroughly identified in breeding geese. A basic range is still lacking for investigating and optimizing dietary VE formulation. In addition, an exceptionally high intake of VE (approximately 2000 IU/kg) was indicated to harm animal fertility, as reported in a study on sheep [17]. We noted that some managers have tried to supply geese diets with a high quantity of VE to maintain late laying performance, but achieved decreased egg production and fertility. Based on such issues, we hypothesized that an appropriate range of VE supplementation (40 IU/kg to 200 IU/kg) would improve the reproductive performance of geese. Thus, further exploration needs to be conducted on how a high dose of VE inclusion induces a decline in the performance of breeding geese. Clarifying the effects of dietary VE levels (VE deficiency to high dose VE) on reproductive performance is necessary and practically meaningful for geese production. 

Egg characteristics are closely related to a bird’s age, diet, and nutrient composition [16]. Dietary VE supplementation alters the utilization of protein, lipids, vitamins, and minerals in breeding poultry and affects the nutrient deposition in eggshells, yolk, and albumen [18]. An increased resistance of chicken embryonic and postnatal tissues to oxidative stress and the establishment of chick immune defense was observed to result from increased VE that was transferred from the maternal diet to the yolk [14,19,20]. In addition, a very high level of dietary VE (10,000 IU/kg and 20,000 IU/kg) could severely decrease the transfer of maternal carotenoids to egg yolk [21]. However, little research has been reported on the effect of dietary VE on geese egg characteristics, especially the antioxidant capacity in egg components; this warrants further investigation. 

Dietary VE protects semen quality by preventing the breakdown of polyunsaturated fatty acids from oxidation, thereby improving fertility functions in males [15,22]. The addition of VE acts as a chain-breaking antioxidant to defend cellular membranes against reactive oxygen species and free radical generation [23,24]. Meanwhile, VE indirectly strengthens a body’s antioxidant defense by enhancing the activities of antioxidant enzymes, including superoxide dismutase (SOD), glutathione peroxidase (GSH-Px), catalase (CAT), and glutathione (GSH) [25,26]. For female geese, however, whether and how dietary VE addition can improve reproductive performance by enhancing antioxidant capacity is unclear and needs to be proven by further correlation analysis. Maternal breeders that received VE supplementation were also found to maintain immune status by increasing serum immunoglobulins and cytokines concentrations and promoting immune organ development [13,16]. Nevertheless, either positive or negative antioxidant and immunity-related potential resulting from VE supplementation need more scientific evaluation in breeding geese. 

This study aimed to tentatively evaluate the physiological response of geese to dietary VE (from VE deficiency to high-dose VE) during the late laying period and explore a basic VE range for further studies of the nutrient requirements and optimal doses in breeding geese. Therefore, four dietary levels of VE (0, 40, 200, and 2000 IU/kg) were selected from previous studies [5,16,17], representing VE deficiency, basic-dose VE, middle-dose VE, and high-dose VE, respectively. The evaluation focused on the effects of VE on reproductive performance, egg characteristics, antioxidant capacity, and immune status. 

## 2. Materials and Methods

### 2.1. Birds, Experimental Design, and Management

This study used 480 female and 96 male Jiangnan White breeding geese at 48 weeks of age. The geese were hatched from the same batch offered by a commercial hatchery (Jiangsu Lihua Animal Husbandry Co., Ltd., Changzhou, China). All procedures of this study were permitted by the Institutional Animal Care and Use Committee (IACUC) of the Yangzhou University Animal Experiments Ethics Committee, with permit number SYXK (Su) IACUC 2020-0910.

All breeding geese had the same initial laying rate and similar body weight and were assigned to four treatment groups consisting of four replicates (pens) of 30 female and six male birds each. The four groups were fed a basal diet supplemented with 0 IU/kg, 40 IU/kg, 200 IU/kg, and 2000 IU/kg dietary VE in DL-α-tocopherol acetate form (effective content ≥ 50.0%) purchased from a commercial manufacturer (Xincheng Co., Ltd., Xinchang, China). The basal diet was formulated to meet the nutrient requirements of geese, according to the NRC (1994) [27] and prior research results [28] from our laboratory. All of the experimental diets were pelleted and analyzed for α-tocopherol concentration. The composition and nutrient levels of the diets are listed in Table 1. The feeding experiment was conducted in the experimental pasture of Yangzhou University from December 2020 to the following January. The laying period lasted 6 weeks, from 48 to 54 weeks of age, after 2 weeks of adaptive feeding. During the feeding period, the geese were kept in separate hard-plastic-floored pens that were laid 70 cm above the ground, and they mated naturally. Each pen was equipped with a wooden nest of the same size for laying eggs. The geese were housed under 14 h (4:00 a.m. to 6:00 p.m.) of light per day, and the room temperature was 2 ± 3 °C. All of the geese had access to feed and water ad libitum throughout the trial. The health status and feed intake of the geese were checked daily. No goose died during the experiment.

### 2.2. Determination of Reproductive Performance

Geese eggs were collected, marked, and weighed daily at 5:00 a.m. and 2:00 p.m. Eggs with damage, deformity, frosted eggshell surface, and extreme weight (≤120 g or ≥180 g) were unqualified. At 53 weeks of age, the qualified eggs from each group were collected for 5 consecutive days before hatching. The results were expressed as the mean of four replicates per group and calculated as follows:

Laying rate (%) = Total number of eggs/Total number of geese ÷ Total number of days × 100%;(1)

Qualified egg rate (%) = Total number of qualified eggs/Total number of geese × 100%;(2)

Egg fertility (%) = Total number of fertilized eggs/Total number of qualified collected eggs × 100%;(3)

Hatchability of fertilized egg (%) = Total number of hatched eggs/Total number of fertilized eggs × 100%;(4)

Healthy gosling rate (%) = Total number of healthy chicks/Total number of hatched eggs.(5)

### 2.3. Determination of Egg Characteristics

At the end of the experiment, three qualified eggs were collected randomly from each replicate (48 eggs in total). The length and width of the eggs were measured using an FHK egg shape determinator (Fujihira Industry Co., Ltd., Tokyo, Japan), and the egg shape index was calculated (i.e., length:width). After weighing, the eggshell, albumen, and yolk were separated and weighed. The eggshell percentage, the albumen percentage, and the yolk percentage were calculated. Eggshell thickness (μm) was determined by measuring the average thickness taken at three locations on the eggshell (sharp end, blunt end, and middle section of the eggshell). Yolk color was measured using the Yolk color chart (Robotmation Co., Ltd., Tokyo, Japan) at three places (blunt, equatorial, and sharp regions), with the average used for analyses. Yolk samples were taken and stored at −80 °C to determine the α-tocopherol concentration and antioxidant capacity indices.

### 2.4. Sample Collection and Procedure

At the end of 54 weeks of age, all geese were weighed individually after fasting for 6 h. Then, three female breeding geese were randomly selected from each replicate. Blood samples were collected in sterile procoagulant tubes via wing venipuncture, centrifuged immediately at 4500 rpm at 4 °C for 15 min to obtain plasma, then stored at −80 °C for further analyses. Following this, the geese were exsanguinated by severing the jugular vein and carotid artery on one side of the neck. After bleeding and eviscerating, the percentage content of the heart, liver, spleen, and ovary in live body weight was calculated. Follicles were separated from the ovaries and classified according to diameter (DM; i.e., DM ≤ 3 mm, 3 mm < DM ≤ 6 mm, 6 mm < DM ≤ 9 mm, 9 mm < DM ≤ 12 mm, and DM > 12 mm) and counted. Liver and ovarian tissue samples were removed carefully and stored at −80 °C to determine the antioxidant index. The final result of each index for further determination was expressed as the mean value of three geese (or three eggs) from each replicate.

### 2.5. Determination of VE (α-Tocopherol) in Diets and Egg Yolk

Feed samples were smashed twice by an FW-100 grinder (Taisite Instrument Co., Ltd., Tianjing, China). Then, the feed powder was sieved through a 0.28 mm sieve, harvested in a sealing bag, and stored at −20 °C until analysis. Six replicated samples of each diet were prepared with 35 g per replicate. Samples of egg yolk were prepared with 15 g per each egg in the above way after drying with a freeze dryer (SCIENTZ-12N, Xinzhi Freeze Drying Equipment Co., Ltd., Ningbo, China) at −70 °C for 72 h.

Feed and egg yolk extractions were prepared by the method described in the national standard (GB 5009.82-2016) [29]. Briefly, that method was as follows. Sample powder (1.5 g) was added to an anaerobic tube; 6 mL ethanol, 1 mL 10% L-ascorbic acid, and 2 mL KOH solution (KOH/water = 1:1, g/mL) were then added. The tube was filled with nitrogen, sealed, and mixed using a vortex. A water bath (70 °C) was carried out for 30 min, followed by cooling on ice. The saponification solution was transferred to a 50 mL tube with 2% NaCl (20 mL). Ten mL of anhydrous ether was added. The solution was shaken at 2500 rpm for 2 min, then kept still for layering. The upper organic phase was carried out in a 50 mL centrifuge tube. Anhydrous ether (5 mL) was added once again to extract the α-tocopherol. The upper organic phase (extracting solution) was mixed in a 50 mL centrifuge tube from the two extraction processes. Ten mL of water was added to wash the ether. The solution was centrifuged at 2500× *g* at 4 °C for 10 min. Five mL of the upper organic phase was dried with nitrogen, dissolved with methanol to 1 mL, and filtered via membrane (0.45 μm) for further analysis. 

The α-tocopherol content in the feed and muscle extractions was measured by a Waters E2695 high performance liquid chromatography system (HPLC) (Waters Co., Ltd., Milford, CT, USA), equipped with a chromatographic column (Zorbox SB-C18, 250 mm × 4.6 mm, 5 μm) with a working temperature of 30 °C. The mobile phase was methanol/water at 98%: 2%, with a flow rate of 1 mL/min. The detection was carried out at 300 nm wavelength with an injection of 50 μL extraction. The standard curve (1.56, 3.12, 6.24, 15.59, 31.183, and 62.366 μg/mL) and the working curve were established according to GB 5009.82-2016 [29]. The retention time of the α-tocopherol was approximately 21 min to 22 min. The concentration of the α-tocopherol was expressed as μg/g dry matter.

### 2.6. Determination of Plasma Parameters

The α-tocopherol and VA in plasma were extracted by the filter paper method and n-hexane method. In detail, that method was as follows. Plasma, calibrator, and a quality control solution (in the commercial kit from Nuomingzhetian Co. Ltd., Yangzhou, China) were permeated through special filter paper and dried naturally to make a dry circle paper sample with 6 mm DM. The paper sample and 150 μL of 10% methanol solution were added together in a 2 mL EP tube and shake for 10 min. Then, 450 μL of internal standard solution (1%; standard, 800 ng/mL VE in methanol, 800 ng/mL VA in methanol; 300 ng/mL butylated hydroxytoluene in acetonitrile) was added, vortexed for 30 s, and shaken at 2500 rpm for 10 min. One1 mL of n-hexane was added, shaken at 2500 rpm for 10 min, and centrifuged at 12,000 rpm for 10 min. Eight hundred μL of the sample extraction was blown dry with nitrogen at 25 °C (flow rate, 20 L/min) and dissolved with 100 μL of 0.1% formic acid in methanol; then, it was vortexed at 1000 rpm for 5 min and centrifuged at 4000 rpm for 3 min. The prepared sample extractions were stored at 4 °C and determined within 24 h. 

Concentrations of α-tocopherol and VA were together determined by a liquid chromatography-tandem mass spectrometry (LC-MS/MS) system consisting of a Shimadzu LC20AD HPLC (Shimadzu Co. Ltd., Kyoto, Japan) equipped with a chromatographic column (Bonshell C18 Plus, 3 mm × 50 mm, 2.7 μm, 35 °C of working temperature), and an API 3200MD triple quadrupole mass spectrometer (Sciex Pte. Ltd., Toronto, ON, Canada).The mobile phase A was water/formic acid at 1000:1. The mobile phase B was methanol/formic acid at 1000:1. The flow rate was 1 mL/min. The injection of extraction was 50 μL. The MS adopted atmospheric pressure chemical ionization ion source (APCI) and positive ion MRM scanning analysis mode. The atomizing gas was 60 psi; the curtain gas was 20 psi; the collision gas was 5 psi; the needle current was 5 mA; and the ion source temperature was 400 °C. The ratio between the actual peak area of the sample to be tested and the internal standard peak area was substituted into the standard curve equation (based on peaks of seven standards) to calculate the concentration of the compound to be tested in the sample to be tested. 

Determination of plasma Vitamin D_3_ (VD_3_) concentration was similarly extracted by the filter paper method and n-hexane method and used the same LC-MS/MS system, as follows. The major difference was in nitrogen blowing (60 °C, 0.02 MPa, 10 min). Then, derivative agent PTAD was added for derivatization, and shaken at 500 rpm for 30 min. After that, sample extractions were transferred to a protein filter plate and filtered by shaking at 4000 rpm for 2 min. The mobile phase A was water/formic acid at 1000:1. The mobile phase B was methanol/formic acid at 1000:1. The flow rate was 0.5 mL/min. The injection of extraction was 20 μL. The working temperature of the chromatographic column was 40 °C. The MS atomizing gas flow was 3 L/min; the dry gas flow was 10 L/min; the heating gas flow was 10 L/min; the interface temperature was 300 °C; the DL temperature was 250 °C; the heating block temperature was 400 °C; and thecollision-induced dissociation (CID) gas was 230 kPa. 

Plasma reproductive hormones, including follicle-stimulating hormone (FSH, cat. No. H101-1-2), luteinizing hormone (LH, cat. No. H206-1-2), estradiol (E_2_, cat. No. H102-1), progesterone (P, cat. No. H089), prolactin (PRL, cat. No. H095-1-1), either plasma immunoglobulins including immunoglobulin A (IgA, cat. No. H108-1-2), immunoglobulin M (IgM, cat. No. H109-1-2), and immunoglobulin G (IgG, cat. No. H106-1-1), were determined by the method of enzyme-linked immunosorbent assay (ELISA), using commercial kits following the protocols provided by the manufacturer (Nanjing Jiancheng Bioengineering Institute, Nanjing, China) as described by Amevor et al. [30] and Sun et al. [31]. 

### 2.7. Determination of Antioxidant Enzymes Activity

Each liver, ovarian tissue, and egg yolk sample was accurately weighed to 0.5 g, and a nine-fold volume of specified diluent by weight was added. The samples were homogenized under ice bath conditions by a JXFSTPRP-I-02 fast homogenizer (Shanghai Jingxin Co., Ltd., Shanghai, China) until no particles were visible in the homogenate solution (approximately 60 s). The prepared homogenized solution was centrifuged at 2500 rpm at 4 °C for 10 min to remove debris. One portion of the acquired supernatant was retained for analysis, and the others were immediately stored at −80 °C for further determination. All tissue samples were determined for total antioxidant capacity (T-AOC, cat. No. A015-2-1), superoxide dismutase activity (SOD, cat. No. A001-1), catalase (CAT, cat. No. A007-1-2), glutathione peroxidase activity (GSH-Px, cat. No. A005-1-2), as described by Sun et al. [31], using the commercial assay kits following the protocols of the manufacturer (Nanjing Jiancheng Bioengineering Institute, Nanjing, China). By using the specific assay kits (Nanjing Jiancheng Bioengineering Institute, Nanjing, China), all yolk samples were determined for the total antioxidant capacity (T-AOC, cat. No. A015-2-1), the superoxide dismutase activity (SOD, cat. No. A001-1), and glutathione (GSH, cat. No. A006-2-1), as described by Sun et al. [31] and Amevor et al. [30] Total protein (TP, cat. No. A045-3-2) was determined by the method of bicinchoninic acid (the BCA method). The results were normalized against the TP concentration of each sample for intersample comparison.

### 2.8. Lipid Peroxidation Analysis

Lipid peroxidation was expressed as malondialdehyde (MDA) concentration. Liver, ovarian tissue, and egg yolk were determined by a commercial assay kit (MDA, cat. No. A003-1) following the protocols of the manufacturer (Nanjing Jiancheng Bioengineering Institute, Nanjing, China) as described by Amevor et al. [30]. Briefly, the MDA level of each tissue supernatant was measured by the method of thiobarbituric acid (TBA). The MDA-TBA mixture produced during the reaction of MDA in tissue with TBA was measured at 535 nm by a UV-1780 ultraviolet spectrophotometer (Shimadzu Suzhou Instruments Mfg. Co., Ltd., Suzhou, China). The results were normalized against the TP concentration of each sample for intersample comparison.

### 2.9. Statistical Analysis

All of the data were initially processed using Excel 2019 and analyzed using SPSS 20.0 (SPSS Inc., Chicago, IL, USA). One-way ANOVA with a post hoc test was used to elucidate significant differences. All of the data were checked for normality. Duncan’s test was used for multiple comparisons when a significant difference was detected. The correlation between the variables was analyzed with bivariate Pearson’s correlation coefficients. The results were expressed as means and SEM. The difference was significant when *p* ≤ 0.05, and extremely significant when *p* < 0.01.

## 3. Results

### 3.1. Reproductive Performance

Table 2 shows the effects of dietary VE supplementation on laying performance from 49 to 54 weeks of age. Similar body weights of geese at 54 weeks of age were observed among all groups (*p* > 0.05). Dietary VE supplementation of 40 IU/kg, 200 IU/kg, and 2000 IU/kg significantly increased the qualified egg rate and hatchability of fertilized eggs, compared with 0 IU/kg (*p* < 0.05). Both 0 IU/kg and 2000 IU/kg supplementation negatively reduced egg fertility, compared with 40 IU/kg supplementation and 200 IU/kg supplementation, respectively (*p* = 0.002). Laying rates tended to be highest in geese that were fed 200 IU/kg VE, compared with all other groups (*p* = 0.073). In addition, all groups exhibited similar healthy gosling rates (*p* > 0.05).

### 3.2. Follicular Development

Table 3 shows the number of follicles graded according to different diameters in geese at 54 weeks of age. A trend in the increase of the ovary index was found with increased dietary VE supplementation (*p* = 0.063). Compared with the 0 IU/kg group, the VE supplementation of 200 IU/kg significantly increased the number of follicles, with a DM ≤ 3 mm (*p* = 0.015) and the total number of follicles (*p* = 0.021). Geese that were fed a dietary VE supplementation of 2000 IU/kg exhibited the highest number of follicles, with a DM > 3 mm and ≤ 6 mm, compared with VE supplementation of 40 IU/kg (*p* = 0.035). The numbers of follicles with a DM > 6 mm (i.e., 6 mm < DM ≤ 9 mm, 9 mm < DM ≤ 12 mm, DM > 12 mm) were similar among all groups (*p* > 0.05).

### 3.3. Plasma Reproductive Hormones

Table 4 shows the plasma concentrations of reproductive hormones, including FSH, LH, PRL, E_2_, and P, in geese at 54 weeks of age. Dietary VE supplementation of 40 IU/kg, 200 IU/kg, and 2000 IU/kg significantly increased plasma concentrations of FSH, compared with dietary VE supplementation of 0 IU/kg (*p* = 0.046). Compared with 0 and 2000 IU/kg, geese fed with dietary VE supplementations of 40 IU/kg and 200 IU/kg exhibited significantly higher plasma concentrations of E_2_ (*p* = 0.019) and *p* (*p* = 0.042). In addition, plasma concentrations of LH and PRL were unaffected by dietary VE supplementation (*p* > 0.05).

### 3.4. Egg Characteristics 

Table 5 shows the egg quality and yolk VE deposition in geese determined at 54 weeks of age. With an increase in dietary VE supplementation, the yolk color depth (*p* < 0.001) and VE content (*p* < 0.001) increased significantly. Eggs in groups with VE supplementation of 40 IU/kg, 200 IU/kg, and 2000 IU/kg had significantly higher albumen rates than the 0 IU/kg group (*p* = 0.001). In addition, dietary VE supplementation did not affect the egg shape index, eggshell thickness, or yolk rate (*p* > 0.05).

### 3.5. Plasma Vitamin Concentration

Table 6 shows the plasma concentrations of vitamins, including VE, VA, and VD_3_, in geese at 54 weeks of age. The plasma concentration of VE increased significantly by increasing dietary VE supplementation, up to 2000 IU/kg (*p* < 0.001). The plasma concentrations of VD_3_ in the 40 IU/kg, 200 IU/kg, and 2000 IU/kg groups were similar, and were all higher than that in the 0 IU/kg group (*p* < 0.001). The plasma concentrations of VA were unaffected by dietary VE supplementation (*p* > 0.05).

### 3.6. Antioxidant Capacity 

Table 7 shows the MDA concentration and antioxidant enzyme activity of the liver and ovary in geese at 54 weeks of age. In the liver, the MDA concentration significantly decreased with increasing dietary VE supplementation from 0 IU/kg to 200 IU/kg, whereas the difference between the groups with 0 and 2000 IU/kg VE supplementation was insignificant (*p* = 0.003). Compared with 0 IU/kg of supplementation, the T-AOC activity significantly increased with VE supplementation from 40 IU/kg to 200 IU/kg, then decreased at 2000 IU/kg (*p* = 0.001). The SOD and GSH-Px activities significantly increased with VE supplementation of 40 IU/kg and 200 IU/kg, compared with VE supplementation of 0 IU/kg and 2000 IU/kg (*p* < 0.001). In addition, the CAT activities were unaffected by treatments (*p* > 0.05). 

In the ovary, the MDA concentration decreased significantly with increasing VE supplementation from 0 IU/kg to 200 IU/kg, but inclined to 0 IU/kg at 2000 IU/kg (*p* < 0.001). Compared with VE supplementation of 0 IU/kg, the T-AOC activities significantly increased in the groups with VE supplementation of 40 IU/kg to 2000 IU/kg, with no difference among these treatments (*p* < 0.001). The SOD activities significantly increased with VE supplementation from 0 IU/kg to 2000 IU/kg (*p* < 0.001). However, the CAT activities were unaffected by treatments (*p* > 0.05).

The MDA concentration and antioxidant enzyme activities of egg yolk in geese were determined at 54 weeks of age. Dietary VE supplementation of 200 IU/kg and 2000 IU/kg significantly decreased the MDA concentration, compared with VE supplementation of 0 IU/kg (*p* = 0.005), whereas the differences between the groups with VE supplementation of 0 IU/kg to 40 IU/kg, 40 IU/kg to 2000 IU/kg, and 200 IU/kg to 2000 IU/kg were not significant. The T-AOC activities in the groups with VE supplementation of 200 IU/kg and 2000 IU/kg were significantly higher than that in the control group, whereas the differences between groups with VE supplementation of 0 IU/kg to 40 IU/kg, 40 IU/kg to 200 IU/kg, and 40 IU/kg to 2000 IU/kg were not significant (*p* = 0.028). The SOD activities in the groups with VE supplementation of 40 IU/kg, 200 IU/kg, and 2000 IU/kg were significantly higher than that in the control group, whereas the difference between the groups with VE supplementation of 200 IU/kg and 2000 IU/kg was insignificant (*p* < 0.001). However, there was no difference in GSH activities among all treatments (*p* = 0.062). 

### 3.7. Immune Status

Table 8 shows the spleen index and plasma content of immunoglobulins, including IgA, IgM, and IgG, in geese at 54 weeks of age. Compared with the group with VE supplements of 0 IU/kg, geese that were fed 40 IU/kg, 200 IU/kg, and 2000 IU/kg of dietary VE supplementation had a higher spleen index (*p* < 0.05). The plasma content of IgA (*p* = 0.012) and IgG (*p* < 0.001) significantly increased with the increasing levels of VE supplementation, from 0 IU/kg to 2000 IU/kg (*p* < 0.05). The plasma content of IgM was unaffected by all treatments (*p* > 0.05). 

### 3.8. Correlation Analysis

Table 9 shows that the laying rate was positively correlated with the activities of T-AOC (*p* < 0.05), CAT (*p* < 0.05), and GSH-Px (*p* < 0.01) in the liver. The qualified egg rate was positively correlated with the T-AOC activity in the liver (*p* < 0.05). Egg fertility was negatively correlated with the MDA concentration in the liver (*p* < 0.01) and ovary (*p* < 0.01), and was positively correlated with the activities of T-AOC (*p* < 0.01), SOD (*p* < 0.05), and GSH-Px (*p* < 0.05) in the liver, as well as SOD (*p* < 0.05) and GSH-Px (*p* < 0.05) in the ovary.

Table 10 shows that the VE deposition was negatively correlated with the MDA concentration in the egg yolk (*p* < 0.01), and was positively correlated with the activity of SOD (*p* < 0.01). The hatchability of fertilized egg was positively correlated with the activity of GSH (*p* < 0.05)

Table 11 shows that the qualified egg rate was positively correlated with the plasma content of IgA (*p* < 0.01) and IgM (*p* < 0.01). The healthy gosling rate was positively correlated with the spleen index (*p* < 0.01).

## 4. Discussion

### 4.1. Reproductive Performance 

The qualification of eggs determines the number of incubated eggs and thereby affects offspring yield. In this study, the number of qualified eggs decreased dramatically when the geese diet was not supplemented with VE. The presence of substandard eggs, specifically eggs that are broken (fragile), deformed, frosted, soft-shelled, or contaminated with blood or feces, indicates abnormality or inflammation of the breeding goose’s reproductive tract or gonads, which is particularly evident in the late laying period. The results suggest that dietary VE can alleviate the reproductive decline of female geese at the end of egg production and thereby maintain normal egg formation. 

The current laying rate was similar to that (24.93 to 31.07%) reported by Zhang et al. [10], who supplied 40 IU/kg VE to Wulong breeding geese diets supplemented with *Bacillus subtilis*. However, both fertility and hatchability were lower than those set out above. This could be due to different goose breeds, and/or that the current geese were in the late laying period. Early studies proved that VE supplementation enhanced egg fertility and hatchability in chickens [32,33]. Urso et al. [14] found that the hatchability of breeding poultry that were fed 120 IU/kg VE was higher than that of breeding poultry that were fed 30 IU/kg VE after a 7 week feeding period. Abedi et al. [7] also observed that 30 IU/kg to 240 IU/kg of dietary VE significantly increased the hatchability of fertilized eggs and decreased embryonic mortality in Japanese quails, compared with zero addition of VE. Likewise, the current study showed increased egg fertility and hatchability with increased dietary VE levels up to 200 IU/kg; no significant difference was found with VE supplementation of between 40 IU/kg and 200 IU/kg. In addition, egg fertility was further reduced by a high addition of VE at 2000 IU/kg. By extrapolation, these findings preliminarily suggest that VE supplementation of 40 IU/kg to 200 IU/kg can be a basic range for further studies of precise VE demand in breeding geese.

### 4.2. Follicular Development

Follicular development on the ovary is essential in maintaining egg production in poultry. In the current study, supplementing VE up to 200 IU/kg enhanced ovarian function, which was manifested in the slightly enlarged ovary index and the increased number of developing follicles. However, the laying rate was not influenced by different levels of VE supplementation, which was similarly reported by Amevor et al. [34]. Follicles were heavily occluded at the early period of yolk enrichment, resulting in a sharp decrease in the number of pre-ovulatory follicles. Barreto et al. [35] and Shahriar et al. [36] also observed no changes in laying rate when VE (up to 250 IU/kg) was included in the broiler diets. We speculated that a certain VE feeding dosage was insufficient to alter the rhythmic physiological activity of ovulation that is dominated by the genetic background of geese. 

### 4.3. Plasma Reproductive Hormones

The FSH is a major hormone that regulates follicular development and selection. It promotes the secretion of P and E_2_ from ovarian granulosa and endometrial cells in geese [37]. In the current study, 200 IU/kg of dietary VE supplementation increased the plasma FSH, E_2_, and P concentrations, further supporting the improvement of follicular development. Similarly, in Japanese quails [7], adding 120 IU/kg VE increased the serum concentration of E_2_ and increased the weight of developing follicles. LH is the main hormone that drives ovulation in mature follicles. As follicles approach maturation, their responsiveness to FSH diminishes and their responsiveness to LH increases [38]. The results of the current study indicated that dietary VE may not affect the ovulatory process. Notably, avian reproductive hormone secretion is temporally rhythmic [39] and can only be used to aid in the interpretation of follicular development and in the ovulation process. 

### 4.4. Egg Characteristics

The current study observed higher egg weight, higher yolk rate, and similar eggshell thickness, compared with those in Wulong breeding geese [10]. Although 40 IU/kg VE was included in both diets, the difference in egg characteristics could be induced primarily according to geese breeds. Maternal dietary VE was suggested to improve egg quality by enhancing the eggshell quality and changing the egg components [6,40,41]. Amevor et al. [16] reported that dietary 200 IU/kg VE (vs. 0 IU/kg) increased egg yolk weight, yolk height, yolk diameter, albumen height, and the Haugh unit after a 10 week trial from 52 weeks of age. As the Haugh unit is an indicator of protein content in albumen, dietary VE was proved to be capable of improving egg protein content. In addition, the yolk color depth was enhanced by the combination of quercetin (0.4 g/kg) and VE, which may be due to the synergistic effects of their pigment compounds (xanthophyll and carotenoid). In agreement with the above, the current study observed an increased albumen percentage and yolk color depth. This improvement can be retained after the high intake of VE up to 2000 IU/kg. However, other research indicated that dietary VE could not affect fresh egg quality [14,42,43,44,45]. The controversy may stem from different bird species, ages, feeding periods, and dosages of VE in diets. 

### 4.5. Plasma Vitamin Concentration

In the current study, the elevation of dietary VE significantly increased plasma VE concentration. As reported by Surai [46], deposition of VE from diet occurs simultaneously in the blood, liver, kidney, heart, and gonads, which can consist of antioxidant defense throughout the reproductive process. Interestingly, the plasma VD_3_ concentration decreased when VE was supplemented. As indicated by the improved egg qualification, the formation of eggshells increased the consumption of VD_3_ and calcium, thereby maintaining a low level of VD_3_ in the blood. However, the exact mechanism needs to be identified via further investigation. 

### 4.6. Antioxidant Capacity 

In the study of Jiang et al. [5], supplementation with 200 IU/kg VE (vs. 0 IU/kg) from 40 to 63 weeks of age decreased the MDA concentration and increased the activities of SOD and GSH-Px in the serum of layers. A reduced serum MDA concentration was also observed when hens received 250 IU/kg VE (vs. 0 IU/kg) from 44 to 56 weeks of age [43]. Yang et al. [47] reported that hens that were fed 100 IU/kg VE (vs. 0 IU/kg) for 12 weeks showed an increase in the activities of SOD and T-AOC in the ovary an ad decrease in MDA concentration at 71 weeks of age. Likewise, the present result shows an enhanced antioxidant status in both the liver and the ovary with VE supplementation of 40 IU/kg and 200 IU/kg VE. Specifically, the positive correlation further indicated that dietary VE could increase egg fertility via such enhancement. The antioxidant effect of VE could improve ovarian function and result in a better quality of mature ova. Moreover, that earlier study explained that VE could benefit the redox environment of the reproductive tract in breeding poultry, particularly the sperm stored site, thereby improving the probability of egg fertility [48]. 

However, the high addition of VE (2000 IU/kg vs. 200 IU/kg) reduced antioxidant enzyme activity and increased MDA concentration in tissue. Eder et al. [49] reported that excess dietary VE (1000 mg and 10,000 mg all-rac-α-tocopherol acetate/kg diets) lowered the activities of SOD, CAT, and GSH-Px in the erythrocytes of rats that were fed salmon oil after 8 weeks. To the best of our knowledge, excessive VE will instead become an oxidant when VE does not have sufficient vitamin C to reduce. More practically, superabundant VE could play an essential role in protecting breeding geese and their eggs from oxidant stress induced by high-temperature exposure and bacterial or toxin infection [12,50,51,52,53]. Hoehler and Marquardt [54] supplied 1000 IU/kg VE in chicken portions containing ochratoxin A and T-2 toxin and found decreased MDA in the liver (vs. 0 IU/kg). The results of the current study suggest that both VE deficiency and its high intake could negatively impact the organ antioxidant capacity associated with the egg fertility of geese. 

In this study, maternal VE was shown to be delivered effectively into the egg yolk during yolk formation. As correlatively observed from the deposition of VE, the elevated activities of T-AOC, SOD, and GSH, and the decreased MDA concentration, further indicated that dietary VE improved the antioxidant capacity of yolk. Previous studies reported similar improvements in laying hens that received 200 IU/kg VE (vs. 0 IU/kg) [5,16,45,47]. Accordingly, under the protection of the antioxidant system formed with the participation of VE, pigment oxidation could weaken, thereby deepening the egg yolk coloration [6]. During egg incubation, VE was shown to be effectively transferred from the egg yolk to the developing embryo [55,56]. As shown by the positive correlation in this study, the provided antioxidant defense could be responsible for embryonic development and improved hatchability with dosage effect [14,57]. In addition, in the current study, we did not observe any adverse effect of high intake of VE (2000 IU/kg) on the antioxidant capacity of yolk, egg quality, or hatchability. It was possible that VE was not over-deposited in the geese eggs. As Surai et al. [58] explained, there are species–specific differences in the efficiency of VE transfer from the feed to the egg yolk, with laying hens being more efficient than turkeys, ducks, or geese.

### 4.7. Immune Status

The spleen participates widely in humoral and cellular immunity as the largest peripheral immune organ in geese [8]. Nutritional research suggested that the enlargement of immune organs without visible pathological damage commonly represents a positive immune response to feed ingredients, which indicates that the immune function was enhanced [8,59,60]. The present results showed that dietary VE supplementation enlarged the spleen index, thereby improving the immune status of breeding geese. The same improvement was explicitly reported in laying hens [16,53]. Furthermore, the positive correlation between the spleen index and a healthy gosling rate also indicates that dietary VE can maintain the health of offspring by improving the maternal immune status. It is possible that the goslings received more maternal antibodies from the parental generation [13,14,19].

Previous studies [59,61] indicated that when the spleens of healthy geese were enlarged by dietary nutrients, this was potentially accompanied by an improvement in blood immune parameters. Sun et al. [31] reported that dietary 12 to 48 IU/kg VE upregulated serum concentrations of IgA and IgG and downregulated interferon-γ (INF-γ) in goslings at 28 days of age. In aging laying hens, the supplementation of 200 IU/kg VE (vs. 0 IU/kg) increased the serum content of immunoglobulins (IgA, IgG, and IgM) and decreased INF-γ and Interleukin-2 (IL-2) [16], as well as in layers that were challenged with *Salmonella Enteritidis* [53]. The mucosal immune system of the reproductive tract is an important barrier against pathogen invasion. Following synthesis in plasmacyte, IgA binds to secretory components within the cell membrane of epithelial cells to form secretory IgA (sIgA), which acts by translocating across mucosal epithelial cells to the lumen [61]. Meanwhile, IgM and IgG act as the most abundant antibodies in the blood and are mass-produced to fight against foreign invaders and reduce proinflammatory factors [62]. Similarly, the current results show an increase in the plasma content of IgA and IgG, and a trend in the increase of IgM, in which a high intake of VE achieved the optimal immune parameters. The positive correlation between the plasma immunoglobulin content and the qualified egg rate may support the conclusion that dietary VE can protect the reproductive system of breeding geese from inflammation or decline by enhancing the body’s immune function. 

## 5. Conclusions

Dietary VE deficiency hampered reproductive performance in breeding geese in the late laying period, possibly through the reduction of VE deposition, antioxidant capacity, and immune status. Supplemental doses of VE up to 200 IU/kg improved the qualified egg rate by increasing immunoglobulin production. In addition, a dose of 200 IU/kg improved egg fertility and hatchability by enhancing antioxidant enzyme activity and decreasing MDA in the liver and ovary. Egg hatchability improved by VE may also be explained by more VE deposition and higher antioxidant enzyme activity promoted by VE retention. Moreover, the 2000 IU/kg dose of VE caused an overdose reaction that lowered liver antioxidant status and impacted egg fertility adversely. In summary, both VE deficiency and high-dose VE (2000 IU/kg) reduced reproductive performance, whereas a dose of 200 IU/kg VE achieved optimal fertility, possibly by enhancing antioxidant capacity and immune status in breeding geese. Further studies are needed to investigate the precise VE requirement in the range of 40 IU/kg to 200 IU/kg during the late laying period.

## Figures and Tables

**Table 1:** Composition and nutrient levels of basal diets (as-fed basis).

Items	Vitamin E/(IU/kg)			
	0	40	200	2000
Ingredient (%)				
Rice	63.90			
Maize	9.80			
Soybean meal (CP, 46%) ^1^	12.40			
Puffed soybeans	3.20			
Limestone	7.70			
Calcium bisphosphate	0.59			
Methionine	0.23			
Lysine	0.10			
Choline chloride	0.08			
Premix ^2^	2.00			
Nutrient levels ^3^				
Metabolizable energy (MJ/kg) ^4^	10.90			
Crude protein (%)	14.00			
Crude fiber (%)	5.70			
Crude ash (%)	2.50			
Calcium (%)	3.15			
Total phosphorus (%)	0.53			
Available phosphorus (%)	0.30			
Total lysine (%)	0.75			
Digestible lysine (%)	0.65			
Vitamin E (IU/kg) ^5^	11.36	48.79	207.13	1 992.5

^1^ CP, crude protein. ^2^ One kilogram of premix contained the following: 600,000 IU retinol, 400,000 IU rachitasterol, 112.5 mg coagulation vitamin, 110 mg thiamine, 487.5 mg riboflavin, 187.5 mg pyridoxine, 1.375 mg cobalamin, 1.875 g nicotinic acid, 750 mg pantothenic acid, 70 mg folic acid, 4.75 mg biotin, 3 g Fe (ferrous sulfate), 0.5 g Cu (copper sulfate), 5 g Mn (manganese sulfate), 4.5 g Zn (zinc sulfate), 25 mg I (potassium iodide), and 15 mg Se (sodium selenite). ^3^ Analyzed values except for metabolizable energy. ^4^ Metabolizable energy was calculated from the ingredient apparent metabolizable energy (AME) values for chickens. ^5^ α-tocopherol measured via the HPLC method. Mean value of six random samples of each diet.

**Table 2:** Effects of dietary vitamin E supplementation on reproductive performance in breeding geese.

Items ^1^	Vitamin E/(IU/kg)				SEM	*p*-Value
	0	40	200	2000		
BW at 54 wks of age/g	5025	4912	5147	4868	64.1	0.435
Laying rate/%	27.68	26.79	30.05	27.21	0.50	0.073
Qualified egg rate/%	89.64 ^b^	94.61 ^a^	94.55 ^a^	94.74 ^a^	0.82	0.049
Egg fertility/%	64.59 ^C^	71.42 ^AB^	75.33 ^A^	67.47 ^BC^	1.20	0.002
Hatchability of fertilized egg/%	86.23 ^b^	90.87 ^a^	88.96 ^ab^	89.81 ^a^	0.62	0.037
Healthy gosling rate/%	93.67	93.41	94.43	94.57	0.38	0.674

^a,b,c^ Means with different superscripts within each row are significantly different (*p* < 0.05). ^A,B,C^ Means with different superscripts within each row are extremely significantly different (*p* < 0.01). ^1^ BW, body weight; wks, weeks. Each value represents the mean of the four replicates in each group.

**Table 3:** Effects of dietary vitamin E supplementation on follicular development in breeding geese.

Items ^1^	Vitamin E/(IU/kg)				SEM	*p*-Value
	0	40	200	2000		
Ovary index/%	0.26	0.37	0.37	0.38	0.02	0.063
DM ≤ 3 mm	37.42 ^B^	46.16 ^AB^	52.83 ^A^	46.40 ^AB^	1.88	0.015
3 mm < DM ≤ 6 mm	11.28 ^ab^	9.17 ^b^	12.83 ^ab^	15.01 ^a^	0.73	0.035
6 mm < DM ≤ 9 mm	7.14	9.33	8.84	7.40	0.89	0.799
9 mm <DM ≤ 12 mm	5.43	2.34	3.67	2.00	0.54	0.076
DM > 12 mm	3.50	4.00	4.80	4.40	0.19	0.080
Total follicles number	64.29 ^b^	71.01 ^ab^	82.17 ^a^	72.70 ^ab^	2.27	0.021

^a,b,c^ Means with different superscripts within each row are significantly different (*p* < 0.05). ^A,B,C^ Means with different superscripts within each row are extremely significantly different (*p* < 0.01). ^1^ DM, diameter. The ovary index (%) = the weight of ovary (g)/the BW (g) × 100%. Each value represents the mean of the four replicates (two geese per replicate) in each group.

**Table 4:** Effects of dietary vitamin E supplementation on plasma reproductive hormones in breeding geese ^1^.

Items	Vitamin E/(IU/kg)				SEM	*p*-Value
	0	40	200	2000		
FSH/(mIU/mL)	6.78 ^b^	7.19 ^ab^	7.90 ^a^	7.44 ^ab^	0.15	0.046
LH/(mIU/mL)	33.71	32.15	37.43	35.09	0.73	0.060
PRL/(ng/mL)	9.51	9.09	9.16	9.34	0.14	0.766
E_2_/(pg/mL)	136.06 ^b^	165.11 ^ab^	176.54 ^a^	143.47 ^b^	5.44	0.019
P/(ng/mL)	1.11 ^b^	1.34 ^a^	1.29 ^a^	1.22 ^ab^	0.03	0.042

^a,b^ Means with different superscripts within each row are significantly different (*p* < 0.05). ^1^ FSH, follicle stimulation hormone; LH, luteinizing hormone; PRL, prolactin; E_2_, estradiol; P, progesterone. Each value represents the mean of the four replicates (two geese per replicate) in each group.

**Table 5:** Effects of dietary vitamin E supplementation on egg characteristics in breeding geese.

Items	Vitamin E/(IU/kg)				SEM	*p*-Value
	0	40	200	2000		
Average egg weight/g	147.58	148.68	147.98	146.32	1.51	0.959
Egg shape index	1.42	1.39	1.41	1.42	0.01	0.645
Eggshell thickness/μm	476.06	472.55	463.16	473.76	5.42	0.847
Eggshell rate/%	10.35	10.03	10.13	10.12	0.11	0.727
Yolk color depth	1.45 ^D^	2.09 ^C^	3.07 ^B^	3.5 ^A^	0.19	<0.001
Yolk rate/%	32.88	31.03	32.65	32.22	0.38	0.297
Albumen rate/%	50.18 ^B^	54.68 ^A^	53.57 ^A^	54.56 ^A^	0.51	0.001
Average egg weight/g	147.58	148.68	147.98	146.32	1.51	0.959
Yolk VE content/(μg/g) ^1^	32.70 ^C^	61.64 ^C^	129.88 ^B^	215.52 ^A^	16.94	<0.001

^A,B,C,D^ Means with different superscripts within each row are extremely significantly different (*p* < 0.01). ^1^ VE, vitamin E. Each value represents the mean of the four replicates (three eggs per replicate) in each group.

**Table 6:** Effects of dietary vitamin E supplementation on plasma concentration of vitamin E, A, and D_3_ in breeding geese ^1^.

Items/(ng/mL)	Vitamin E/(IU/kg)				SEM	*p*-Value
	0	40	200	2000		
Vitamin E	3792.6 ^D^	6821.2 ^C^	13,442.8 ^B^	42,920.6 ^A^	3158.2	<0.001
Vitamin A	744.86	769.10	724.01	723.98	22.06	0.881
Vitamin D_3_	13.25 ^A^	6.75 ^B^	8.38 ^B^	5.72 ^B^	0.61	<0.001

^A,B,C,D^ Means with different superscripts within each row are extremely significantly different (*p* < 0.01). ^1^ Each value represents the mean of the four replicates (two geese per replicate) in each group.

**Table 7:** Effects of dietary vitamin E supplementation on antioxidant capacity in breeding geese ^1^.

Items	Vitamin E/(IU/kg)				SEM	*p*-Value
	0	40	200	2000		
Liver						
MDA/(nmol/mg prot)	1.47 ^A^	1.02 ^BC^	0.77 ^C^	1.22 ^AB^	0.08	0.003
T-AOC/(mmol/g prot)	5.36 ^C^	8.32 ^AB^	9.11 ^A^	7.08 ^B^	0.43	0.001
SOD/(U/g prot)	174.51 ^C^	217.96 ^B^	315.05 ^A^	163.08 ^C^	16.32	<0.001
CAT/(U/mg prot)	53.89	51.71	56.47	50.43	1.02	0.162
GSH-Px/(U/mg prot)	122.41 ^BC^	135.48 ^B^	187.36 ^A^	115.59 ^C^	7.81	<0.001
Ovary						
MDA/(nmol/mg prot)	2.53 ^A^	2.32 ^B^	2.06 ^C^	2.44 ^AB^	0.05	<0.001
T-AOC/(mmol/g prot)	3.43 ^b^	4.36 ^a^	4.48 ^a^	4.54 ^a^	0.16	0.019
SOD/(U/g prot)	154.12 ^C^	171.11 ^C^	198.61 ^B^	237.85 ^A^	8.65	<0.001
CAT/(U/mg prot)	12.61 ^C^	11.85 ^C^	18.27 ^A^	15.68 ^B^	0.71	<0.001
GSH-Px/(U/mg prot)	116.52	129.69	123.94	130.78	2.37	0.120
Egg yolk						
MDA/(nmol/mg prot)	20.56 ^A^	18.26 ^AB^	14.79 ^C^	15.58 ^BC^	0.71	0.005
T-AOC/(mmol/g prot)	2.03 ^b^	2.24 ^ab^	2.40 ^a^	2.45 ^a^	0.06	0.028
SOD/(U/g prot)	35.69 ^C^	47.78 ^B^	63.82 ^A^	59.32 ^A^	2.74	<0.001
GSH/(mg/g prot)	32.76	35.83	41.37	38.89	1.23	0.062

^a,b^ Means with different superscripts within each row are significantly different (*p* < 0.05). ^A,B,C^ Means with different superscripts within each row are extremely significantly different (*p* < 0.01). ^1^ prot, protein; MDA, malondialdehyde; T-AOC, total antioxidant capacity; SOD, total superoxide dismutase; GSH-Px, glutathione peroxidase; CAT, catalase; GSH, glutathione. For tissue, each value represents the mean of the four replicates (two geese per replicate) in each group. For egg yolk, each value represents the mean of the four replicates (three eggs per replicate) in each group.

**Table 8:** Effects of dietary vitamin E supplementation on immune status in breeding geese ^1^.

Items	Vitamin E (IU/kg)				SEM	*p*-Value
	0	40	200	2000		
Spleen index/%	0.04 ^b^	0.05 ^a^	0.05 ^a^	0.05 ^a^	0.01	0.048
IgA/(μg/mL)	219.70 ^b^	265.78 ^ab^	295.83 ^a^	301.29 ^a^	10.31	0.012
IgM/(μg/mL)	1 225.1	1 299.4	1 388.4	1 409.5	29.32	0.092
IgG/(μg/mL)	1 995.3 ^B^	2 420.7 ^AB^	2 564.1 ^AB^	2 886.3 ^A^	81.50	<0.001

^a,b^ Means with different superscripts within each row are significantly different (*p* < 0.05). ^A,B^ Means with different superscripts within each row are extremely significantly different (*p* < 0.01). ^1^ IgA, immunoglobulin A; IgM, immunoglobulin M; IgG, immunoglobulin G. The spleen index (%) = The weight of spleen (g)/The BW (g) × 100%. Each value represents the mean of the four replicates (two geese per replicate) in each group.

**Table 9:** Correlation analysis of reproductive performance index and antioxidant capacity index in liver and ovary ^1^.

Items	MDA		T-AOC		SOD		CAT		GSH-Px	
	Liver	Ovary	Liver	Ovary	Liver	Ovary	Liver	Ovary	Liver	Ovary
Laying rate	−0.248	−0.493	0.531 *	0.166	0.495	0.314	0.532 *	−0.258	0.637 **	0.453
Qualified egg rate	−0.343	−0.357	0.501 *	0.201	0.292	0.366	0.069	0.183	0.284	0.273
Egg fertility	−0.682 **	−0.638 **	0.530 *	0.320	0.669 **	0.557 *	0.141	0.065	0.549 *	0.574 *
Hatchability of fertilized egg	−0.099	−0.242	0.421	0.422	0.150	0.274	0.053	0.269	0.029	0.139
Healthy gosling rate	−0.358	−0.167	−0.133	0.408	−0.131	0.123	−0.238	0.276	−0.112	−0.045

* The statistical difference (*p* < 0.05). ** Highly significant difference (*p* < 0.01). ^1^ MDA, malondialdehyde; T-AOC, total antioxidant capacity; SOD, total superoxide dismutase; GSH-Px, glutathione peroxidase; CAT, catalase.

**Table 10:** Correlation analysis of vitamin E (VE) content, hatchability of fertilized egg, and antioxidant capacity index in egg yolk ^1^.

Items	MDA	T-AOC	SOD	GSH
Yolk VE content	−0.583 **	0.415	0.709 **	0.443 *
Hatchability of fertilized egg	0.393	0.338	−0.204	0.522 *

* The statistical difference (*p* ≤ 0.05). ** Highly significant difference (*p* < 0.01). ^1^ MDA, malondialdehyde; T-AOC, total antioxidant capacity; SOD, total superoxide dismutase; GSH, glutathione.

**Table 11:** Correlation analysis of reproductive performance index and immune index ^1^.

Items	Spleen Index	IgA	IgG	IgM
Laying rate	0.058	0.216	−0.125	0.190
Qualified egg rate	−0.180	0.693 **	0.383	0.713 **
Egg fertility	0.377	0.191	0.289	0.259
Hatchability of fertilized egg	−0.169	0.191	−0.105	0.369
Healthy gosling rate	0.499 *	0.109	0.468	0.095

* The statistical difference (*p* ≤ 0.05). ** Highly significant difference (*p* < 0.01). ^1^ IgA, immunoglobulin A; IgM, immunoglobulin M; IgG, immunoglobulin G.

## Data Availability

The data presented in this study are available on request from the corresponding Author.
